# Thrombosis of an Arteria Lusoria with Secondary Subclavian Steal Syndrome and Swallowing Difficulties

**DOI:** 10.5334/jbr-btr.821

**Published:** 2015-12-30

**Authors:** P. Vlummens, B. Houthoofd, W. Janssens

**Affiliations:** 1 Department of hematology, Ghent University Hospital, De Pintelaan 185, 9000 Ghent, Belgium; 2 Department of radiology and medical imaging, St Vincentius General Hospital, 9800 Deinze, Belgium; 3 Department of geriatrics, Ghent University Hospital, De Pintelaan 185, 9000 Ghent, Belgium

**Keywords:** artery, thrombosis, dysphagia

## Abstract

We report a case of an arteria lusoria causing swallowing difficulties known as dysphagia lusoria. Although the presence of an arteria lusoria is quite common, dysphagia lusoria is relatively rare. Interestingly, our patient also presented with a concurrent aneurysmal dilatation, known as a Kommerell’s diverticulum, at the aortic origin. Complete thrombosis of the artery and flow reversal in the right cervical artery resulting in an asymptomatic subclavian steal syndrome was also seen. No underlying primary pro-thrombotic defects were identified but due to the presence of locally advanced prostate cancer, a paraneoplastic phenomenon was suspected.

A 87-year-old man with no apparent medical history was admitted to the geriatric ward due to malaise which had been persisting for several months. He reported anorexia and intermittent dysphagia. A routine chest X-ray was negative and subsequently contrast enhanced CT imaging of the thorax was performed, revealing the presence of a thrombotic arteria lusoria with an aneurysmal dilatation at its aortic origin (Figure 1). The arteria lusoria also applied external pressure when passing alongside the esophagus, resulting in stasis of saliva and swallowing difficulties (Figure 2). Blood flow to the right upper extremity was provided through the right vertebral artery, resulting in an anatomical subclavian steal phenomenon (Figures 3A/B and 4A/B). Peripheral pulmonary emboli could also be seen in the absence of deep venous thrombosis. An extensive hematological work-up was performed with a full coagulation panel as arterial and venous events were identified but no pro-thrombotic defects could be identified. Further investigations revealed the presence of locally advanced prostate cancer so the presence of a paraneoplastic pro-thrombotic state was suspected. Anticoagulants were started but the patient refused any further vascular or oncologic treatment.

**Figure 1:**
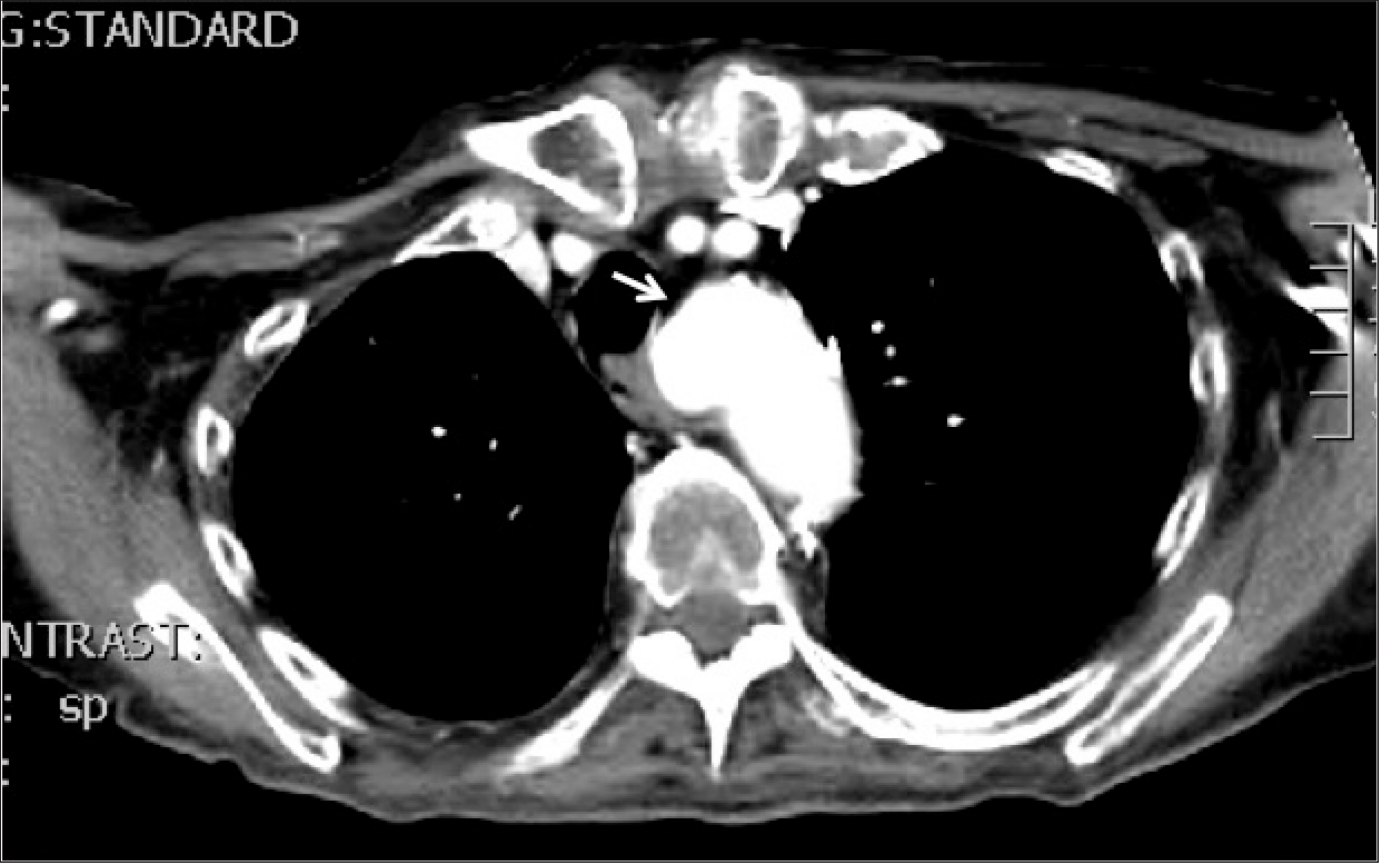
Kommerell’s diverticulum (arrow), ie. an aneurysmal dilation of the onset of the aberrant retro-esophageal and retrotracheal traversing right subclavian artery, the arteria lusoria.

**Figure 2:**
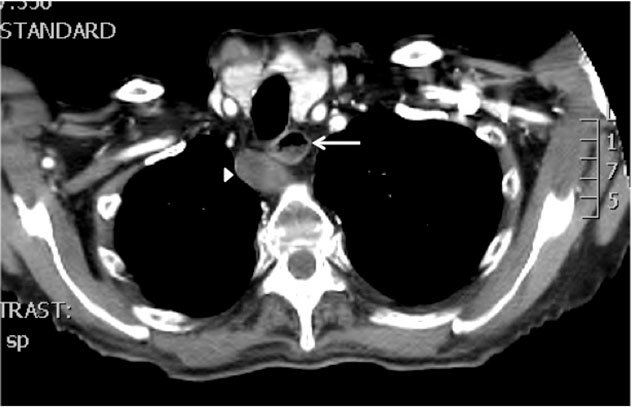
Thrombosed, enlarged arteria lusoria (arrowhead) with compression and slight anterior deviation of the esophagus. There is a readily apparent fluid level visible in the esophagus just above the course of the arteria lusoria (arrow), indicative of compression and representing stasis of ingested food and mucus.

**Figure 3:**
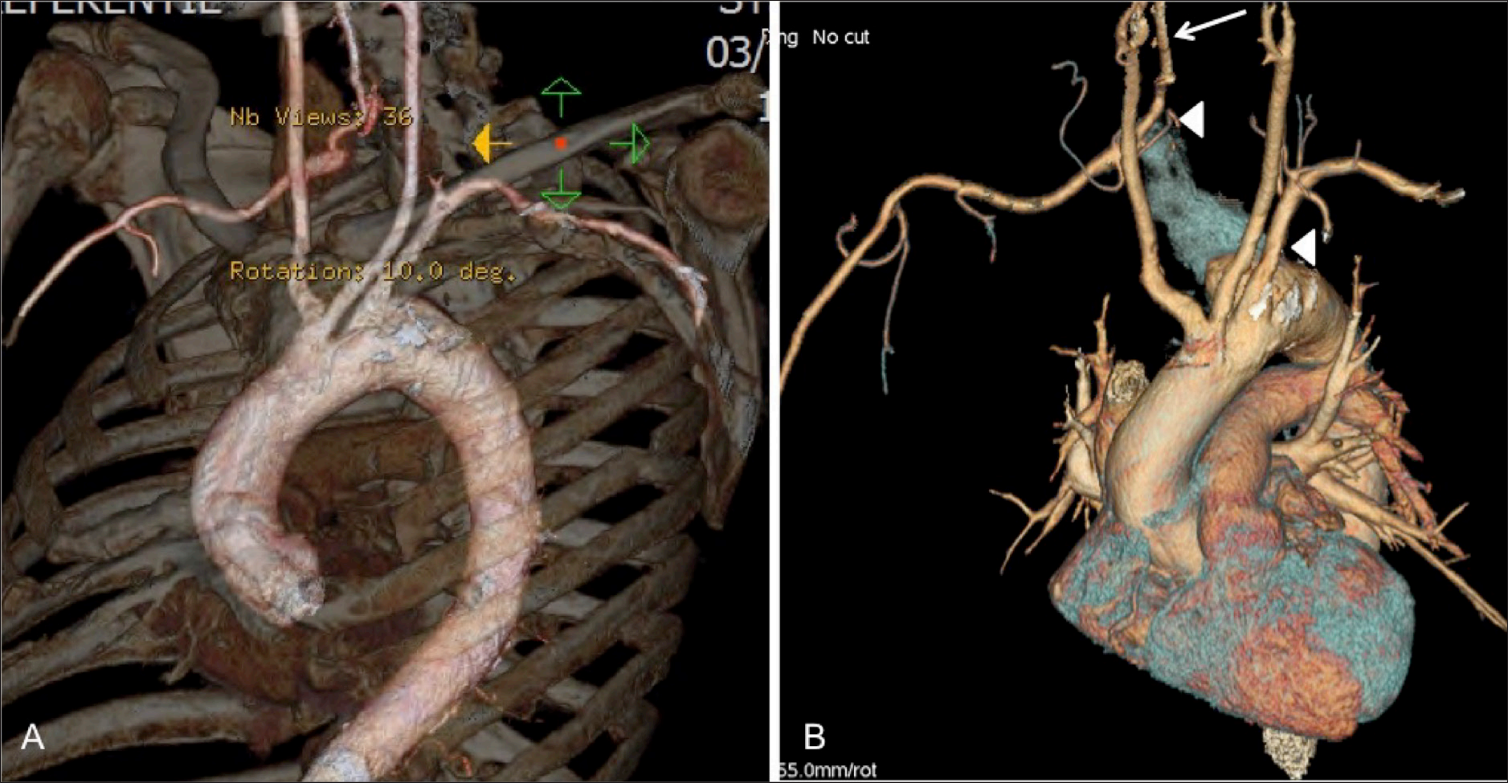
(A) Volume rendered image of the aortic arch and supra-aortic arteries with bone reference; (B) Volume rendered image of the heart and aortic arch with supra-aortic vessels: these images show the aberrant origin of the right subclavian artery, posterior to the left subclavian artery. Furthermore, the proximal right subclavian artery is “grayed out” (between arrowheads), ie. no longer patent, and there is visible retrograde flow filling of the more peripheral right subclavian via the right vertebral artery (subclavian steal phenomenon) (arrow).

**Figure 4:**
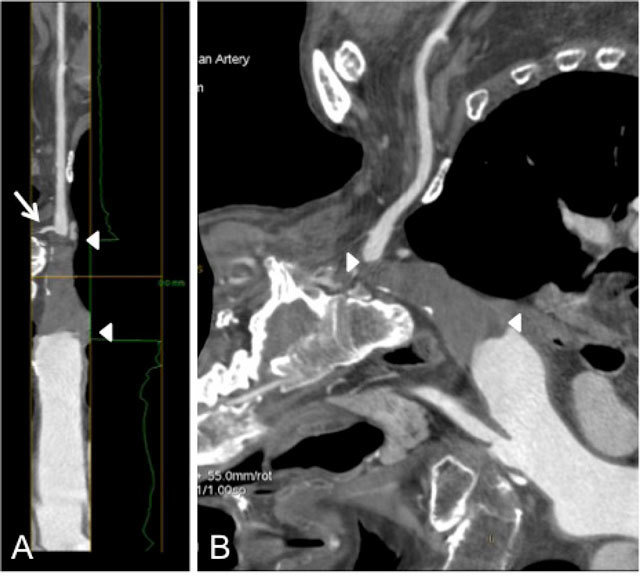
(A) Lumen view of the right subclavian artery, showing complete thrombosis (between arrowheads) of the proximal part; contrast opacification of the more peripheral part via subclavian steal through the right vertebral artery (arrow); (B) Curved multiplanar recontruction of the aortic arch and proximally thrombosed right subclavian artery.

## Discussion

**Figure 5:**
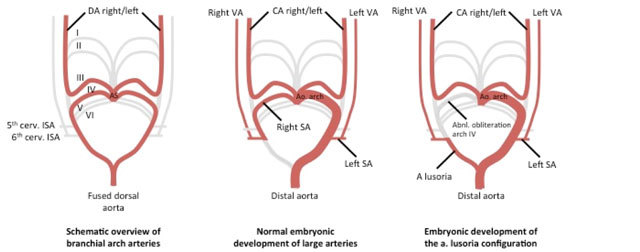
Schematic overview of the developmental anatomy underlying the generation of the arteria lusoria configuration. Due to abnormal obliteration of the 4th pharyngeal arch, the right subclavian artery originates from the right dorsal aorta, thus finally arising from the proximal descending aorta instead of the ascending aorta. (*I to VI: pharyngeal arches 1 to 6, respectively; Ao: aortic; AS: aortic sinus; CA: carotid artery; cerv: cervical; ISA: intersegmental artery; SA: subclavian artery; VA: vertebral artery*).

Of all congenital anomalies of the aortic arch an abberant subclavian artery, also known as an arteria lusoria, is the most common one being present in 0.3–2% of the general population [1,2]. It is caused by disturbances of development of the fourth pharyngeal arch. During the course of embryonic growth six pairs of pharyngeal arches develop, giving rise to different vascular, neurological and musculoskeletal structures of the head and neck region. Under normal circumstances the subclavian artery is derived from the remodeling of the right-sided fourth pharyngeal arch and the segment of the right-sided dorsal aorta distal to this arch, along with the ipsilateral sixth cervical intersegmental artery (Figure 5). In case of an arteria lusoria however, the embryonic development of the right-sided fourth pharyngeal arch is defunct, typically affecting the portion of the right-sided dorsal aorta distal to the sixth cervical intersegmental artery. As such, the aberrant vessel is not connected to the ascending aorta or proximal aortic arch, but to the descending aorta through the right dorsal aorta (Figure 5). In this embryonic configuration the right subclavian artery arises from the proximal descending aorta instead of the ascending aorta and must travel upwards and to the right either behind the esophagus (80–84%), between the esophagus and trachea (12,7–15%) or less frequently anterior to the trachea (4,2–5%) [3]. In 30–60% of cases an aneurysmal dilatation of the onset of the arteria lusoria at the aortic arch, known as a Kommerell’s diverticulum, can also be seen and it is thought to be a remainder of the primitive right aortic arch, which normally regresses during embryogenesis [4]. An arteria lusoria frequently presents itself as an asymptomatic and isolated defect, thus being diagnosed accidentaly during routine investigations or autopsy in most cases. In few cases however, it exerts local compression of the esophagus and causes so called dysphagia lusoria, as was first described by Bayford in 1794 [5]. The mean age of presentation is between 40 and 48 years and patients with mild to moderate symptoms are mainly treated conservatively with surgical reconstruction only being indicated in those patients presenting with severe or persisting symptoms [6,7]. The type of surgical procedure depends of the age of the patient and the effect of the artery on its surrounding tissues. In children ligation of the right subclavian artery is sometimes performed but this approach is not suitable in adults as there is a high risk for developing claudication of the right arm and/or symptomatic subclavian steal syndrome. In adult patients other surgical methods have been described. As open vascular surgery is associated with high mortality alongside elevated rates of neurological events, it has been gradually replaced by hybrid techniques encompassing endovascular occlusion with subsequent open revascularisation [3,8]. Large studies focusing on the outcome of patients treated using these strategies are however still lacking.

In our case, surgical treatment options were explored and discussed as our patient presented with clear symptoms of anorexia and intermittent dysphagia. However, due to the presence of significant co-morbidity on one hand and the fact that our patient declined further therapy on the other hand, a fully conservative treatment approach was chosen.

Interestingly, we report the first patient who seems to present himself with a total occlusion of the abberant artery, together with an asymptomatic subclavian steal phenomenon. This was quite intriguing from a clinical point-of-view as higher rates of thrombosis at the level of the arteria lusoria have not yet been reported in the current literature. As venous and arterial thrombotic events were identified, other pathophysiological mechanisms were explored through an extensive work-up of the coagulation system which succesfully excluded diffuse intravascular coagulation, factor V Leiden, factor II mutations, anti-phospolipid syndrome, elevated levels of factor VIII, protein C/S deficiency or hyperhomocysteinemia. As subsequent investigations revealed the presence of a locally advanced stage of prostate cancer, we suspected the presence of a paraneoplastic pro-thrombotic state. Disorders of hemostasis are known to be commonplace in patients with prostate cancer through hyper-expression of tissue factor, cancer pro-coagulant, and platelet-activating factor, inducing the release of pro-thrombotic and pro-fibrinolytic substances into the bloodstream [9]. As it was the patient’s wish to decline further investigations or invasive therapy, a dissection at the level of the artery could unfortunately not be ruled out, although its presence is unlikely due to the chronic clinical course. Finally, anticoagulants were started and further supportive therapy initiated.

## Conclusion

This case stresses the importance of considering the possible presence of an arteria lusoria in the differential diagnosis of patients with chronic swallowing difficulties, as it can lead to significant morbidity when undiagnosed. CT-based techniques are pivotal as they provide vital information on the presence of aneurysmal formation, thrombi or compression of surrounding structures, as clearly illustrated in this case. The abberant artery can be missed when only endoscopic techniques are used to explore the patient’s complaints, further stressing the importance of radiologic imaging techniques. As higher levels of thombosis at the level of the arteria lusoria have not yet been reported in the current literature, we advise further investigations to exclude complications i.e. dissection of the vessel, underlying coagulation defects or the presence of a paraneoplastic pro-thrombotic state, when thrombosis of the abberant vessel is observed.

## Competing Interests

The authors declare that they have no competing interests.
